# Endocannabinoid System and Synaptic Plasticity: Implications for Emotional Responses

**DOI:** 10.1155/2007/52908

**Published:** 2007-06-19

**Authors:** María-Paz Viveros, Eva-María Marco, Ricardo Llorente, Meritxell López-Gallardo

**Affiliations:** Departamento de Fisiología (Fisiología Animal II), Facultad de Biología, Universidad Complutense, 28040 Madrid, Spain

## Abstract

The endocannabinoid system has been involved in the regulation of anxiety, and proposed as an inhibitory modulator of neuronal, behavioral and adrenocortical responses to stressful stimuli. Brain regions such as the amygdala, hippocampus and cortex, which are directly involved in the regulation of emotional behavior, contain high densities of cannabinoid CB1 receptors. Mutant mice lacking CB1 receptors show anxiogenic and depressive-like behaviors as well as an altered hypothalamus pituitary adrenal axis activity, whereas enhancement of endocannabinoid signaling produces anxiolytic and antidepressant-like effects. Genetic and pharmacological approaches also support an involvement of endocannabinoids in extinction of aversive memories. Thus, the endocannabinoid system appears to play a pivotal role in the regulation of emotional states. Endocannabinoids have emerged as mediators of short- and long-term synaptic plasticity in diverse brain structures. Despite the fact that most of the studies on this field have been performed using in vitro models, endocannabinoid-mediated plasticity might be considered as a plausible candidate underlying some of the diverse physiological functions of the endogenous cannabinoid system, including developmental, affective and cognitive processes. In this paper, we will focus on the functional relevance of endocannabinoid-mediated plasticity within the framework of emotional responses. Alterations of the endocannabinoid system may constitute an important factor in the aetiology of certain neuropsychiatric disorders, and, in turn, enhancers of endocannabinoid signaling could represent a potential therapeutical tool in the treatment of both anxiety and depressive symptoms.

## 1. INTRODUCTION

Fear is an adaptive component of the acute stress response to potentially dangerous stimuli which threaten the integrity of the individual. However, when disproportional in intensity, chronic, irreversible, and/or not associated with any actual risk, it constitutes a maladaptive response and may be symptomatic of an anxiety-related neuropsychiatric disorder such as generalized anxiety, phobia, or post-traumatic stress disorder (PTSD), among others. A diversity of mechanisms, including GABAergic, serotonergic, and noradrenergic systems, appears to be involved in the regulation of anxious states which may contribute to an appropriate emotional response to aversive events [1]. In the recent years, an increasing interest in the endocannabinoid system has arisen as part of the complex circuitry that regulates anxiety and as a crucial mediator of emotional learning. Brain distribution of cannabinoid CB1 receptors is consistent with an involvement of this system in the regulation of emotional reactivity. Indeed, CB1 receptors are highly expressed in brain structures such as the amygdala, hippocampus, anterior cingulate cortex, and prefrontal cortex [2–8], key regions in the regulation of emotional responses. Moreover, the cannabinoid CB1 agonist CP 55,940 increased Fos immunoreactivity in brain structures known to be involved in anxiety and fear-related responses such as the central nucleus of the amygdala, the periaqueductal gray, and the paraventricular nucleus (PVN) of the hypothalamus [9].

Depression is a mood disorder in which the prevailing emotional mood is distorted or inappropriate to the circumstances. There are important links between chronic stress and depression. Upon exposure to acute stressful stimuli, the organism initiates a series of neuroendocrine short-term responses that are beneficial in terms of adaptation. However, exposure to chronic, unavoidable situations of stress may have deleterious consequences, including endocrine, emotional, and cognitive alterations associated with neuropsychiatric disorders such as depression. In this context, hyperactivity of the hypothalamus-pituitary-adrenal (HPA) axis with increased glucocorticoids levels appears to be linked to major depression [10, 11]. There is evidence for an involvement of the endocannabinoid system in the regulation of neural, behavioral, and endocrine responses to aversive stimuli [12, 13] and it has been suggested that stress-induced dysregulation of specific components of the endocannabinoid system might be associated with deficits in behavioral flexibility that can be manifested in stress-related disorders such as PTSD and depression [14].

Endocannabinoids have been shown to act as retrograde transmitters at the synaptic level. Though the exact role of retrograde endocannabinoid signaling in vivo is not fully clarified yet, it is likely that by this mechanism endocannabinoids play important roles in synaptic transmission and plasticity, including modulation of emotional responses. Indeed, endocannabinoids have recently emerged as one of the most thoroughly investigated, and widely accepted, classes of retrograde messengers in the brain [15]. Cannabinoid-induced neuroplasticity may underlie diverse physiological functions modulated by the endocannabinoid system, that is, pain [16] and memory [17]. Synaptic plasticity within the amygdala appears to play a crucial role in acquisition, storage, and extinction of aversive memories, basic neural processes that serve adaptive behaviors, and the endocannabinoid system has emerged as a crucial mediator of such neuroplasticity-related phenomena. Marsicano et al. [18, 19] proposed that endocannabinoids facilitate extinction of aversive memories through their selective inhibitory effects on local inhibitory networks in the amygdala, providing evidence for a functional role of endocannabinoid release-based synaptic plasticity. Apart from the amygdala, there are some other brain areas that have been postulated as substrates for cannabinoid-induced neural plasticity such as the hippocampus and the hypothalamus where cannabinoid-dependent synaptic plasticity is involved in the regulation of the stress-response system [17, 20]. Pharmacological modulation of the endocannabinoid system has been proposed as a novel potential therapeutical strategy for the treatment of anxiety disorders and depression [21], and therapeutic interventions directed at normalization of the HPA system [11] might potentially include modulation of endocannabinoid signaling.

## 2. THE ENDOCANNABINOID SYSTEM AND CANNABINOID-RELATED COMPOUNDS

The endocannabinoid system includes the cannabinoid receptors, the endogenous lipid ligands (endocannabinoids), and the enzymatic machinery for their synthesis and inactivation. Endocannabinoids are important neuromodulators that appear to be involved in a plethora of physiological processes such as modulation of nociception, regulation of motor activity, cognitive processes, neuroprotection, immune function and inflammatory responses, antiproliferative actions in tumoral cells, control of cardiovascular system, and neurodevelopment, among others [22–29]. Notably, the endocannabinoid system appears to be critically involved in the maintenance of homeostasis [28, 30]. In this review, we aim to highlight its function as a stress-recovery system.

Endocannabinoids are polyunsaturated fatty acid derivatives. The ethanolamide of arachidonic acid anandamide (AEA) and 2-arachidonoylglycerol (2-AG) are the most studied endocannabinoids and have been implicated in a wide range of physiological and pathological processes. Other molecules such as 2-arachidonyl-glyceryl ether (noladin, 2-AGE), O-arachidonoyl-ethanolamine (virhodamine), and N-arachidonoyl-dopamine (NADA) have been discovered more recently. The anabolic and catabolic pathways for AEA and 2-AG appear to rely on very complex enzymatic cascades and are in the progress of being elucidated. In brief, the enzime N-acylphosphatidylethanolamine-specific phospholipase D (NAPE-PLD) synthesizes AEA from N-arachidonoylphosphatidylethanolamine (NArPE), whereas the diacylglycerol lipase (DAGL) generates 2-AG from diacylglycerol (DAG) substrates. Due to their lipophilic nature, endocannabinoids cannot be stored in vesicles. It is widely accepted that, unlike other mediators, the endocannabinoids are synthesized and released on demand, in response to diverse physiological and pathological stimuli, and appear to exert important actions as retrograde messengers. Endocannabinoid inactivating mechanisms include cellular reuptake and hydrolysis. AEA appears to be taken up by several cell types at least in part via a facilitated transport mechanism, known as the anandamide membrane transporter (AMT), which can also transport 2-AG intracellularly. Though this putative transporter has not been isolated or cloned yet, there are compounds that are considered as inhibitors of cellular uptake. A fatty acid amide hydrolase (FAAH) is the main AEA hydrolase, whereas a monoacylglycerol lipase (MAGL) is critical in degrading 2-AG. It is important to take into consideration that the actions of endocannabinoids are considered to be spatially and temporally restricted. Therefore, the effects of exogenously applied cannabinoids, which lack such selectivity, do not necessarily mimic physiological functions of the endocannabinoid system [26, 28]. Compounds that enhance endocannabinoid signaling by inhibiting endocannabinoid reuptake (e.g., VDM11, OMDM-1, OMDM-2, UCM707) or by degradation (e.g., the FAAH inhibitors URB597, AM374, or N-arachidonoyl-serotonin) are widely used in preclinical studies and appear to have a potential therapeutical interest. A profound discussion of biochemical aspects of the endocannabinoid system is beyond the scope of this paper, but the reader can find comprehensive excellent reviews (e.g., [27, 28, 31–35]) as well as recent papers on specific aspects such as alternative biosynthetic pathways for endocannabinoids [36, 37] and endocannabinoid membrane transport [38].

Cannabinoids mainly exert their pharmacological effects by the activation of specific membrane receptors. Mammalian tissues contain at least two types of cannabinoid receptors, CB1 and CB2, which are metabotropic receptors coupled to G-proteins of the Gi/o type. CB1 receptors are localized mainly in the central nervous system, but are also present in a variety of peripheral tissues; they are among the most abundant and widely distributed G-protein coupled receptors in the brain. Transduction systems include inhibition of adenylyl cyclase and of certain voltage-sensitive calcium channels (predominately, those found presynaptically) and activation of inwardly-rectifying potassium channels and mitogen-activated protein (MAP) kinase [39]. Autoradiographic and immunohistochemical studies have shown that CB1 receptors are expressed in multiple brain areas, including the olfactory bulb, neocortex, pyriform cortex, hippocampus, amygdala, basal ganglia, thalamic and hypothalamic nuclei, cerebellar cortex and brainstem nuclei. In particular, a high density of CB1 receptors is found in cortical and limbic regions associated with emotional responses. The levels of expression vary among the various brain regions and neuronal subpopulations, and there is apparently no strict correlation between levels of expression and receptor functionality. Thus, the activity of cannabinoids at CB1 receptor depends not only on the relative receptor density but also on other factors such as receptor coupling efficiency [2, 28, 40–43]. It has been widely accepted that cannabinoids regulate GABA release by activation of CB1 receptor type, and the highest levels of CB1 cannabinoid receptors are found on the terminals of cholecystokinin-positive GABAergic interneurons [44, 45]. On the other hand, the expression of CB1 receptor in glutamatergic neurons has been vigorously debated in recent years. In fact, some authors proposed that a novel non-CB1/non-CB2 cannabinoid-sensitive receptor could be responsible for the inhibition of glutamatergic neurotransmission [46, 47]. However, it has been now well established that functional cannabinoid CB1 receptors are present on glutamatergic terminals of the hippocampal formation, colocalizing with vesicular glutamate transporter 1 [48], as well as in other cortical areas (see, e.g., [26, 49, 50]). These evidences do not exclude that a non-CB1 receptor might exist in the brain, but there is to date no molecular evidence for such novel receptor.

Cannabinoid CB2 receptors are mostly peripherally located on immunological tissues, and therefore implicated in immunological functions. However, they have also been found within the central nervous system on neurons and glial cells with their expression mainly related to conditions of inflammation [51–53]. More recent immunohistochemical analyses have revealed immunostaining for CB2 receptors in apparent neuronal and glial processes in diverse rat brain areas, including cerebellum and hippocampus [54, 55]. These results change the classical view of peripherally located CB2 receptors and suggest broader functional roles for these receptors.

It has been shown that some of the effects of anandamide are mediated by the transient receptor potential vanilloid type-1 channel (TRPV1), formerly called vanilloid receptor VR1 [39]. These receptors have been traditionally known for their function in sensory nerves where they mediate perception of inflammatory and thermal pain, but they are also expressed within the brain contributing to other important physiological functions. Co-expression of cannabinoid CB1 and TRPV1 receptors was found by using immunofluorescence techniques in diverse brain areas involved in the regulation of emotional responses. In particular, within the hippocampus, CB1/TRPV1 was detected on cell bodies of many pyramidal neurons throughout the CA1–CA3 subfields and in the molecular layer of dentate gyrus [56]. Interestingly, TRPV1 knockout mice (TRPV1-KO) showed less anxiety-related behavior in the light-dark test and in the elevated plus-maze than their wild-type littermates as well as less freezing to a tone after auditory fear conditioning and stress sensitization. TRPV1-KO also showed impaired hippocampus-dependent contextual fear together with a decrease in long-term potentiation (LTP) in the Schaffer collateral-commissural pathway to CA1 hippocampal neurons. These data provide first evidence for fear-promoting effects of TRPV1 with respect to both innate and conditioned fear and for a decisive role of this receptor in synaptic plasticity [57]. Collectively, these findings open new avenues for the study of possible functional relationships between CB1 and TRPV1 receptors, in particular regarding stress, fear, and anxiety responses.

Recently, an additional G-protein-coupled receptor (GPCR) GPR55 has been proposed as a possible new cannabinoid receptor that might play a physiological role in lipid or vascular biology [58].

## 3. BASIC PRINCIPLES OF ENDOCANNABINOID-MEDIATED SYNAPTIC PLASTICITY

One of the most salient features of the nervous system is its plasticity, including structural and functional changes in individual neurons and synapses. This characteristic is present both during brain development and in the adult life. Synaptic plasticity allows changes in the strength and number of synaptic connections between neurons. It is considered as one of the major mechanisms underlying learning and memory and appears to mediate several other functions in the central nervous system. The resulting changes in synaptic efficacy are thought to be crucial in experience-dependent modifications of neural function. A closely related concept is behavioral flexibility that allows an organism to adapt to variable environmental demands and produce adaptive responses.

Given the prominent presynaptic localization of cannabinoid CB1 receptors, together with its mainly inhibitory actions, cannabinoids have been proposed as local retrograde modulators, with an important role in modulating essential physiological functions and contributing in diverse synaptic plasticity phenomena [59–62]. The endocannabinoid system seems to affect neuronal excitability participating in the maintenance of homeostatic conditions in the brain [26, 63, 64]. In this respect, data obtained from conditional CB1 mutant mice suggest that the endocannabinoid system may protect neurons against excessive activity, and consequently against excitotoxicity. Marsicano et al. generated conditional mutant mice that lacked expression of the CB1 receptor in principal forebrain neurons but not in adjacent inhibitory interneurons. In mutant mice, the excitotoxin kainic acid (KA) induced excessive seizures in vivo, and the threshold to KA-induced neuronal excitation in vitro was severely reduced in their hippocampal pyramidal neurons. Moreover, KA administration rapidly raised hippocampal levels of anandamide and induced protective mechanisms in wild-type principal hippocampal neurons, whereas these protective mechanisms could not be triggered in mutant mice. These findings indicate that neural excitability is increased in CB1-deficient mice and that the endocannabinoid system may act as a neuroprotective system against abnormally increased discharge activity [26, 65]. The CB1 receptor-mediated neuroprotective effect in the kainate model is apparently mediated by decrease of excitability of glutamatergic hippocampal neurons [48].

Activation of postsynaptic receptors, at diverse neuronal types, induces the release of endogenous cannabinoid compounds that move backwards across the synapse, until reaching the cannabinoid CB1 receptor, to which they bind, therefore inhibiting further neurotransmitter release. Endocannabinoid-mediated synaptic plasticity can be transient or long lasting and can be found at both excitatory and inhibitory synapses in diverse brain structures. Endocannabinoid-mediated short-term synaptic plasticity includes two electrophysiological phenomena, depolarization-induced suppression of inhibition (DSI), and depolarization-induced suppression of excitation (DSE). DSI is due to a presynaptic action that reduces GABA release, while DSE results from presynaptic inhibition of glutamatergic release. There is also an involvement of the endocannabinoid system in long-term forms of synaptic plasticity. Long-term potentiation (LTP) is a long-lasting increase in the strength of a synapse, while long-term depression (LTD) is a long lasting weakening of synaptic strength. Both are mechanisms of synaptic plasticity that can persist for hours to weeks and have important implications on various forms of learning and memory. Endocannabinoid-induced long-lasting inhibition of neurotransmitter release has been found in diverse brain structures and at both excitatory and inhibitory synapses (for exhaustive discussion of these phenomena, see [15, 26, 64, 66, 67]).

## 4. EFFECTS OF CANNABINOIDS ON ANXIETY-RELATED RESPONSES

The main feature of the recreational use of cannabis is that it produces a euphoriant effect. This “high” can be accompanied by decreased anxiety and increased sociability. However, cannabis can also produce dysphoric reactions, feelings of anxiety, panic, paranoia, and psychosis [68–72]. It is possible that the reasons for this lie on the bidirectional effects of cannabinoids on anxiety, with low doses having anxiolytic, and high doses having anxiogenic-like effects. The previous history of the individual and the environmental context may also critically influence the induced cannabinoid effects. Data from animal models provide further evidence for the complexity of the scenario. Low doses of several cannabinoid receptor agonists, nabilone [73], CP 55,940 [74, 75], and Δ^9^-tetrahydrocannabinol (THC) [76] induced anxiolytic-like effects in both the elevated plus-maze and the light-dark box. In contrast, high doses of the cannabinoid agonist HU-210 produced anxiogenic-like responses in the defensive withdrawal test [77] and enhanced emotional responding to tactile stimulation [78], and mid-high doses of CP 55,940 showed anxiogenic-like effects in the plus-maze [74, 75, 79, 80] and in the social interaction test [81].

It has been shown that exposure to chronic stress enhances the anxiety-like responsiveness to cannabinoids in rats [82], a phenomenon that is also observed in humans. Accordingly, Patel et al. [83] have recently analyzed the interactions between cannabinoids and environmental stress in the regulation of amygdalar activation in mice. The combination of restraint stress and CB1 agonist administration produced robust Fos induction within the central amygdala, indicating a synergistic interaction between environmental stress and CB1 receptor activation. These data suggest that the central amygdala could be an important neural substrate relevant to the context-dependent effects of cannabinoids on emotional/affective responses.

It is worth noting that, in addition to anxiety, there are other behavioral responses, such as motor activity and exploration [75, 80, 81, 84, 85], that are affected by cannabinoid agonists in a biphasic manner. In general, low doses are stimulatory, whereas high doses are inhibitory. Bimodal effects of cannabinoids might be explained by two distinct populations of presynaptic CB1 receptors, with different sensitivities to cannabinoids, particularly WIN 55,212-2 (WIN), located possibly on glutamatergic and GABAergic neurons [26, 86]. The administration of WIN resulted in a biphasic, dose-dependent effect on hippocampal acetylcholine (ACh) release: a low dose and a high dose of the compound induced a transient stimulation and a prolonged inhibition of hippocampal ACh efflux, respectively. These amphidromic responses appeared to involve the same structural entities, Gi-coupled CB1 receptors, but different neuroanatomical sites. The low-dose excitatory effects were mediated in the septum, whereas the high-dose inhibitory effects were mediated locally in hippocampus. Moreover, the stimulatory and the inhibitory effects of the cannabinoid agonist involved activation of dopamine D_1_ and D_2_ receptors, respectively [7]. Local infusion of cannabinoid compounds in specific brain areas might be instrumental to identify neural pathways and neuroanatomically separated CB1 receptor subpopulations that may play distinct roles and mediate opposing actions of cannabinoids, notably, anxiolytic versus anxiogenic effects [87]. This possibility might further explain why elevation of endocannabinoids levels sometimes has effects that are different from those observed with exogenous cannabinoids [26]. An additional hypothesis which might account for the biphasic effects of cannabinoids is the possible differential implication of Gs and Gi proteins in the stimulatory and inhibitory effects, respectively [88]. It would be interesting to test this hypothesis in vivo, in relation to anxiety-related effects.

## 5. ROLE OF THE ENDOCANNABINOID SYSTEM IN THE REGULATION OF ANXIETY

### 5.1. CB1 receptor knockout mice

The development of knockout (KO) mice deficient in CB1(CB1-KO) receptors has provided an excellent tool to evaluate the physiological roles of the endocannabinoid system, and particularly its possible implication in the regulation of anxiety. The CB1-KO mice showed an increase in the aggressive response measured in the resident-intruder test and an anxiogenic-like behavior in the light-dark box, the elevated plus-maze test, and the social interaction test [89, 90]. On the other hand, Marsicano et al. [18] did not find an anxiogenic-like response in the plus-maze in their CB1-KO mice. Discrepancies might be attributed to differences in the genetic background of mutant mice, and also to differences on baseline anxiety levels and to context-dependent stress elicited. In particular, CB1-KO mice exclusively showed an anxiogenic-like behavior under high-stress conditions: light in the plus-maze and unfamiliar environment in the social interaction test [18, 89–91]. An impaired action of anxiolytic drugs, such as bromazepam and buspirone, has been also observed in mutant mice [90]. This latter result suggests that functional integrity of cannabinoid CB1 receptors is necessary to achieve a complete efficacy of anxiolytic drugs, which may have consequences in the treatment of mood-related disorders, including those derived from cannabinoid abuse.

### 5.2. Pharmacological blockade of CB1 receptors

Evidence for an endogenous anxiolytic cannabinoid tone also comes from certain effects of the CB1 receptor antagonist rimonabant (SR141716A). This drug has anxiogenic effects in adult rats submitted to the defensive withdrawal test and the elevated plus-maze [79, 92]. The cannabinoid receptor agonist CP 55,940 reduced ultrasonic vocalization in rat pups separated from their mother, indicating an anxiolytic effect, and rimonabant not only reversed this effect, but also enhanced pup ultrasonic vocalizations when administered alone [93]. These results further support the view that there is an endogenous regulation of emotional states mediated by the cannabinoid system that might be present since early developmental stages. As for CB1-KO animals, certain results obtained in mice following rimonabant administration showed apparently contradictory results since this compound was found to be anxiolytic in the plus-maze [89]. These data may reflect species differences, but it seems likely that environmental context and baseline anxiety levels critically account for at least some of the discrepancies observed in the literature. The context dependency is indirectly supported by the “one-trial sensitization” phenomenon described by Rodgers et al. [94] in the plus-maze. In these experiments, the CB1 receptor antagonist had no behavioral effects in maze-naïve mice, but induced an anxiolytic-like effect in the second trial of the test.

With respect to recent clinical trials, rimonabant has been tested for its possible therapeutical application in obesity and metabolic disorders, and the most frequent adverse events resulting in discontinuation of the drug included depression and anxiety [95–97].

### 5.3. Inhibitors of endocannabinoids inactivation

As indicated above (Section 2), the enzyme FAAH catalyzes the hydrolysis of the endogenous cannabinoid anandamide. Pharmacological blockade of this enzyme by URB597 and URB532 produced anxiolytic-like effects in the elevated zero-maze in adult rats and in the isolation-induced ultrasonic emission test in rat pups. These effects were accompanied by augmented brain levels of anandamide and were prevented by CB1 receptor blockade. Moreover, the anxiolytic actions of URB597 were not accompanied by typical cannabinoid signs of intoxication in rodents such as catalepsy or hypothermia. These results indicate that anandamide participates in the modulation of emotional states and point to FAAH inhibition as an innovative approach to antianxiety therapy [98].

A model has been proposed to explain the possible mechanism by which the AEA-CB1 receptor system may participate in the control of anxious states. Endocannabinoids might be generated in the amygdala in response to the anxiety inducing stimulus, and would, therefore, regulate emotional states by influencing amygdala outputs [99]. This view is supported also by the fact that AEA content in the mouse basolateral amygdala rises when the animal is conditioned to expect a foot shock after hearing a tone [18]. Thus, the endocannabinoid system, and AEA in particular, might be activated in response to anxiogenic situations and this activation could be part of a negative feedback system that limits anxiety [99]. In line with this hypothesis, there are data suggesting a role of endocannabinoid signaling as an inhibitory modulator of behavioral and neuronal responses to aversive stimuli [13] and in the inhibition of stress-induced activation of HPA axis [12] (see next section). A recent paper by Patel and Hillard [100] further supports a crucial role for endocannabinoids in the induction of anxiolytic-like effects. The inhibitor of endocannabinoids metabolism, URB597, produced a linear dose-dependent anxiolytic effect. In turn, AM404 that is considered as an inhibitor of endocannabinoids uptake exerted an action that was more similar to that elicited by direct agonists, with low doses producing anxiolytic effects and the highest dose having no effect [98]. The different profiles of AM404 might be due to the fact that in addition to increasing the endocannabinoid-mediated tone, this compound can also activate TRV1 receptors [101] which, as indicated by the study by Marsh et al. quoted above [57], are also involved in the regulation of anxiety.

Collectively, a majority of evidence suggests the existence of an anxiolytic endocannabinoid tone. The modulatory role of the endocannabinoid system against stress is further supported by studies from Patel et al. [12, 13] indicating that endocannabinoids act as inhibitory modulators of both neuronal and behavioral activations during an acute stress and negatively modulate HPA axis activity (see Section 7).

## 6. CONDITIONED FEAR RESPONSES, AVERSIVE MEMORIES, AND FEAR EXTINCTION

Neurobiological substrates of emotional-based learning have been extensively examined in animal models that allow the study of acquisition, expression, and retention of Pavlovian fear conditioning . In this paradigm, an initially innocuous/neutral stimulus (the to-be conditioned stimulus (CS); e.g., a light, tone, or odor) is paired with an innately aversive unconditioned stimulus (US; e.g., a footshock). Following several pairings, the subject comes to exhibit a conditioned fear response to the CS. Conditioned fear behavioral and physiological responses include changes in heart rate and blood pressure and freezing or cue-induced fear potentiated startle reflex. Excessive fear and anxiety are hallmarks of a variety of disabling neuropsychiatric disorders. Adaptive strategies leading to an appropriate interplay between fear expression and fear extinction are necessary for adequate coping with aversive encounters. In experimental studies like the ones mentioned above, fear inhibition is frequently studied through a procedure in which the previously fear conditioned subject is exposed to the fear-eliciting cue in the absence of any aversive event. This procedure results in a decline in conditioned fear. In other words, repeated presentation of the conditioned stimulus alone leads to extinction of the fearful response. There are clear clinical implications of research on fear extinction. Anxiety- related pathologies such as phobias and post-traumatic stress disorder (PTSD) seem to be disorders of fear dysregulation in which inhibition of fear is absent or insufficient in situations that are patently safe. In the last years, there is an increasing interest in revealing the neural mechanisms of fear inhibition, including the regions in which extinction-related plasticity occurs and the cellular and molecular processes that are implicated in this plasticity-related phenomenon (comprehensive reviews on these mechanisms can be found in [102–105]). In the present section, we will focus on the possible functional implication of the endocannabinoid system.

The use of CB1-KO mice and pharmacological blockade of CB1 receptors have yielded information regarding the involvement of the endocannabinoid system in conditioned fear responses. It has been reported that CB1-KO mice showed strongly impaired short- and long-term extinction in auditory fear-conditioning tests, with unaffected memory acquisition and consolidation. Consistent with this finding, pharmacological blockade of CB1 receptors with rimonabant led to a similar deficit in extinction in wild-type mice [18]. The authors also found that during the extinction protocol (exposure to the tone alone), the levels of endocannabinoids were raised within the basolateral amygdala, a region known to control extinction of aversive memories, both in mutant and normal mice. In subsequent studies, Azad et al. [19] showed that low-frequency stimulation of afferents in the lateral amygdala released endocannabinoids postsynaptically from neurons of the basolateral amygdala of mice, and thereby induced an LTP of inhibitory GABAergic synaptic transmission (LTDi) via a presynaptic mechanism. In turn, lowering inhibitory synaptic transmission significantly increased the amplitude of excitatory synaptic currents in principal neurons of the central nucleus, which is the main output site of the amygdala. LTDi was blocked by rimonabant, abolished in CB1-KO animals, and significantly enhanced in mice lacking FAAH, the anandamide-degrading enzyme [19]. More recently, it has been addressed whether CB1 blockade would similarly disrupt extinction in rats, using fear-potentiated startle as a measure of conditioned fear. The authors further investigated whether pharmacologic augmentation of CB1 activation would lead to enhancements in extinction. The results indicated that rimonabant dose-dependently blocked the extinction of conditioned fear in rats, as it does in mice. Moreover, administration of AM404, an inhibitor of endocannabinoid reuptake, led to a dose-dependent enhancement in extinction and this effect was blocked almost completely by rimonabant, indicating an implication of CB1 receptors. The animals treated with AM404 also showed decreased shock-induced reinstatement of fear, suggesting that this compound may reduce susceptibility to reinstatement of fear [106]. Lin et al. [107] have shown that bilateral infusion of CB1 receptor agonists into the amygdala after memory reactivation blocked reconsolidation of fear memory measured with fear-potentiated startle. These authors proposed that activation of CB1 receptors could facilitate extinction on one hand and block reconsolidation on the other.

Hölter et al. [108] have compared CB1-KO mice with their wild-type controls in an appetitively motivated operant conditioning task including food reward. During the extinction phase, when the positive reinforcement was omitted, control and CB1-KO mice showed a similar decline in accuracy of performance and total number of correct responses, accompanied by an increase in errors of omission [108]. A recent pharmacological study using rimonabant [109] further supports the notion that the cannabinoid CB1 receptor plays a pivotal role in extinction of aversive memories but is not essential for extinction of positively reinforced memories.

It has been claimed that fear conditioning in mice combines both associative and non-associative (sensitization) components and that extinction involves a significant habituation component [110]. In a more recent study, Kamprath et al. [111] have found that CB1-KO mice were severely impaired not only in extinction of the fear response to a tone after fear conditioning, but also in habituation of the fear response to a tone after sensitization with an inescapable footshock. Based on these findings, they have proposed that CB1 receptor might be critically involved in non-associative learning processes (habituation), which would contribute to the decrease in the fear response. A mouse model has been recently proposed that may allow exploring the role of the endocannabinoid system in the associative and non-associative components of fear has been recently proposed [112].

## 7. CANNABINOIDS AND THE HYPOTHALAMUS- PITUITARY-ADRENAL AXIS

An electrophysiological study by Di et al. [20] has revealed that glucocorticoids elicit a rapid, nongenomic suppression of glutamate release onto parvocellular neuroendocrine cells of the hypothalamic paraventricular nucleus (PVN) by stimulating the retrograde release of endocannabinoids that would subsequently activate presynaptic cannabinoid CB1 receptors. By this mechanism, endocannabinoids may be involved in the modulation of a number of peptidergic systems, including CRH. Patel et al. [12] have addressed a role of the endocannabinoid system in the modulation of stress-induced adrenocortical activity in vivo. These authors confirmed previous studies showing that rimonabant was able to increase serum corticosterone concentrations under basal conditions. Moreover, the CB1 receptor antagonist potentiated restraint stress-induced HPA axis activation, whereas pretreatment of mice with either a low dose of the CB1 receptor agonist CP 55,940, the endocannabinoid transport inhibitor AM404, or the FAAH inhibitor URB597 significantly decreased or eliminated restraint-induced corticosterone release. Acute restraint-induced corticosterone release was associated with a decrease in hypothalamic 2-AG content, whereas the attenuation of adrenocortical response observed after prolonged stress was associated with an increase in hypothalamic 2-AG content. In view of the above data, the following speculative model can be suggested: during resting (baseline) conditions, the HPA axis would be tonically inhibited by endocannabinoids via CB1 receptors located in the PVN of the hypothalamus. In this way, the endocannabinoid system might keep under control the stress response. Upon an acute stress exposure, that is, when the stress response is needed, a reduction of endocannabinoids signaling would allow the HPA axis to be activated (disinhibition). If the stress becomes chronic, endocannabinoid levels would increase again to restore a normal homeostasis.

With respect to the effects of exogenous cannabinoid agonists, in general the literature indicates that they exert a dose-dependent effect on adrenocortical activity with high doses increasing corticosterone responses [21, 84, 113]. As previously indicated, high doses of cannabinoids are also anxiogenic. However, we have found that, at certain doses, the effects of cannabinoids on anxiety can be dissociated from their effects on adrenocortical activity. Thus a high dose of the cannabinoid agonist CP 55,940 (75 *μ*g/kg) induced both, anxiogenic-like effects in the plus-maze and stimulation of adrenocortical activity [80]. However, a dose of 50 *μ*g/kg induced an anxiogenic-like effect in the same test, without increasing corticosterone concentrations [113].

As in the case of anxiety, literature regarding HPA axis activity supports the general concept that the pharmacological administration of exogenous cannabinoids may lead to a completely different action when compared with the physiological functions of the endocannabinoid system [26, 28, 30].

## 8. ENDOCANNABINOID SYSTEM AND DEPRESSION

Several lines of evidence suggest that the endocannabinoid system may play a role in the aetiology of depression and could represent a new therapeutic target for its treatment. CB1-KO mice showed altered HPA axis function [90] and a higher sensitivity to exhibit depressive-like responses in the chronic unpredictable mild stress procedure, which suggests an increased susceptibility to develop an anhedonic state [114]. These characteristics together with their heightened anxiety [89, 90] and deficits in extinction of aversive memories [18] have been proposed to be analogous to certain symptoms of melancholic depression [115].

Several cannabinoid compounds have been evaluated in behavioral tests such as the forced swimming test (FST) and the tail-suspension test (TST) that are among the most widely used screening tests of antidepressant potential of novel compounds [116]. In the rat FST, administration of AM404 (endocannabinoid uptake inhibitor) and HU-210, a potent CB1 receptor agonist, induced decreases in immobility (indicative of antidepressant activity) that were blocked by pretreatment with the selective CB1 receptor antagonist AM251. The reduction in immobility induced by the cannabinoid compounds was comparable to that seen with the reference antidepressant desipramine [117]. In turn, the FAAH inhibitor URB597 exerted potent antidepressant-like actions in the mouse TST and the rat FST, and these effects were prevented or attenuated by rimonabant [118].

During the last years, there has been an active investigation on the implications of hippocampal neurogenesis in the pathophysiology and treatment of mood disorders. Preclinical and clinical studies indicate that stress (possibly through the action of elevated glucocorticoids) and depression lead to atrophy and loss of neurons in the adult hippocampus. On the other hand, chronic antidepressant treatment up-regulates hippocampal neurogenesis which could counteract the stress-induced damage [119, 120]. An elegant study by Jiang et al. [121] revealed an important implication of hippocampal neurogenesis in the antidepressant and anxiolytic-like effects of cannabinoid agonists. They showed that both embryonic and adult rat hippocampal neural stem/progenitor cells were immunoreactive for cannabinoid CB1 receptors, indicating that cannabinoids could act on these receptors to regulate neurogenesis. A chronic (but not acute) treatment with the potent synthetic cannabinoid HU210 promoted neurogenesis in the hippocampal dentate gyrus of adult rats and exerted anxiolytic- and antidepressant-like effects. The cannabinoid–induced newborn neurons appeared to be of functional significance, since X-irradiation of the hippocampus blocked both the neurogenic and behavioral effects of chronic HU210 treatment. These evidences strongly suggest that cannabinoid agonists might produce anxiolytic- and antidepressant-like effects by promoting hippocampal neurogenesis. In line with these findings, administration of the endocannabinoids uptake inhibitor AM404 prior to exposure to predator odor stress inhibited both the stress-induced activation of defensive burying and the suppression of cell proliferation in the hippocampus [122], indicating a role for endocannabinoids in the modulation of stress-induced changes in hippocampal cell proliferation.

The efficacy of antidepressants has been linked in part to their ability to reduce the activity of the HPA axis [123]. In view of the above data, it is tempting to speculate that the endocannabinoid system is somehow involved in the action of currently used antidepressant drugs. In favor of this hypothesis, it has been shown that chronic administration of the tricyclic antidepressant desipramine resulted in a significant increase in the density of the cannabinoid CB1 receptor in both hippocampus and hypothalamus as well as in a reduction in swim stress-induced corticosterone secretion and immediate early *c-fos* gene in the medial dorsal parvocellular region of the PVN of the hypothalamus. Moreover, acute treatment with the CB1 receptor antagonist AM251 before exposure to stress occluded the effects of desipramine on corticosterone secretion and neuronal activation [124].

## 9. CONCLUDING REMARKS

During the last few years, the increasing interest in the link between the endocannabinoid system and emotional responses has led to a number of interesting data derived from animal studies. These results may contribute to understand the complex scenario of cannabinoid effects in humans, and to clarify the mechanisms underlying associations between cannabis abuse and mental disorders. Results obtained from transgenic mice lacking CB1 receptors and by using CB1 receptors selective antagonists and inhibitors of endocannabinoids inactivation suggest the existence of an intrinsic endocannabinoid tone which contributes to the regulation of stress responses and anxiety. An adequate endocannabinoid function appears to be necessary for adaptive extinction of aversive memories. The endocannabinoid system might play a pivotal role in maintaining homeostasis, notably with regard to physiological and behavioral responses to acute and prolonged stress. Certain forms of endocannabinoid-dependent synaptic plasticity have been proposed as crucial mechanisms subserving these phenomena. Throughout this review, we have focused on the endocannabinoid system as a major player in the modulation of synaptic transmission and plasticity considering solely interneural communication. However, the critical functional role of glial cells in maintaining a correct brain function and their implications in diverse neuropathological conditions are now clearly recognized. The new concept of the tripartite synapse in which the glial cell (notably astrocytes) plays an active role in the modulation of neurotransmission has recently emerged [125]. Expression of cannabinoid CB1 receptors and endocannabinoid synthesis and release have been observed in different types of glial cells [126, 127]. This “glial endocannabinoid system” may have important physiological and pathological implications [128, 129] and it would be interesting to explore a possible role in the expression of synaptic plasticity in limbic and extra-limbic regions related to stress, fear, and anxiety responses.

Disregulation or malfunctioning of the endocannabinoid system might contribute to the aetiology of anxiety-related disorders and to certain symptoms of melancholic depression. In turn, the endocannabinoid system might constitute an interesting pharmacological target for the development of anti-anxiety and antidepressant therapies.

The involvement of the endocannabinoid system in the regulation of anxiety and its participation in the modulation of behavioral and physiological responses to aversive situations have other obvious implications. Cannabis abuse may be one of the causes disrupting the necessary balance for an appropriate function of the system. There are functional interactions between the endocannabinoid system and other monoaminergic and peptidergic systems also involved in the regulation of emotional responses [113, 130]. Thus, the disruption of the endocannabinoid system as a consequence of cannabis abuse may alter these other neurochemical systems contributing to the development of emotional disorders. In addition to acute aversive emotional reactions to cannabis, the chronic use of this addictive drug may result in mental disturbances and neuropsychiatric disorders. In particular, there are data suggesting that exposure to cannabis derivatives is associated with a higher risk of schizophrenia, depression, and anxiety [68–72, 131, 132]. In this review, we have highlighted the importance of endocannabinoid-based neuroplasticity phenomena in the regulation of neuroendocrine and neurochemical systems implicated in the modulation of emotional responses and extinction of perseverative behaviors and inadaptative aversive memories. Consequently, it is likely that impairment of endocannabinoid-mediated synaptic transmission and plasticity contribute to the expression of at least some aspects of these psychiatric illnesses.
